# Editorial: Multimodal imaging in uveitis

**DOI:** 10.3389/fmed.2022.1011628

**Published:** 2022-09-02

**Authors:** Alex Fonollosa, Ester Carreño, Aniruddha Agarwal

**Affiliations:** ^1^Department of Ophthalmology, Biocruces Bizkaia Health Research Institute, Cruces University Hospital, University of the Basque Country, Barakaldo, Spain; ^2^Department of Ophthalmology, University Hospital Fundación Jimenez Diaz, Madrid, Spain; ^3^Eye Institute, Cleveland Clinic Abu Dhabi, Abu Dhabi, United Arab Emirates

**Keywords:** uveitis, multimodal imaging, angiography, fundus autofluorescence, posterior uveitis, optical coherence tomography, optical coherence tomography-angiography

Ocular imaging is crucial for the proper diagnosis and management of uveitis. Since the introduction of fundus fluorescein angiography (FFA) in the 1960s, new advances in the imaging techniques have allowed a more precise assessment of inflammatory signs in the eye and have been introduced into the routine assessments in clinics. This results in a better understanding of the pathogenesis of diseases and a more accurate monitoring of the inflammatory activity and its complications. The first milestone in imaging techniques was the introduction of FFA. This invasive dye-based technique is essential for objectivating retinal vasculitis and neovascular complications but is also capable of disclosing inflammation in the retina, retinal pigment epithelium (RPE), choroid, or optic nerve. For a better assessment of the choroid, indocyanine green dye-based angiography (ICGA) was subsequently developed which, in the field of ocular inflammation, allowed to objectivate the presence of granulomas or hyperpermeability of the choroidal vessels, among others. An important advancement regarding angiographic techniques has been the development of wide/ultrawide-field imaging, which has made possible to evaluate the periphery in an unprecedented way (1). Introduction of optical coherence tomography (OCT) in the early twentieth century might be considered a revolution in retinal imaging. In a non-invasive and fast manner, inflammatory signs can be observed in the vitreous, in each of the retinal layers, in the RPE, and in the choroid almost as histological slabs. Moreover, automatic quantitative analysis is performed by the device software, allowing an objective monitoring of certain parameters like macular or choroidal thickness (2, 3).

As a matter to remark, enhanced depth imaging-OCT (EDI-OCT) and swept-source-OCT (SS-OCT) technology have a deeper sensitivity and, hence, enable to visualize the choroid with great detail.

Another non-invasive tool with a broad application in posterior uveitis is fundus autofluorescence (FAF). In this case, fluorescence emitted by lipofuscin and other fluorophores upon excitation with specific wavelengths is detected by the device that creates a map of lipofuscin density. FAF is a key tool for the evaluation of entities involving the RPE and outer retina (for instance, the white dot syndromes) where most lipofuscin is generated (4). OCT angiography (OCT-A) is the last tool introduced into the clinics. In addition, OCT-A detects abnormalities in retinal and choroidal blood flow non-invasively and rapidly and allows for an en-face evaluation. Choriocapillaris flow attenuation/deficit due to inflammation in this innermost choroidal layer, or at deeper choroidal levels due to the presence of granulomas, is clear examples of the applications of OCT-A in uveitis (2). Hence, ophthalmologists have a variety of imaging tools (multimodal imaging) at our disposal which enables us to characterize, measure, monitor, and document the different types of inflammatory lesions that occur in posterior uveitis.

In this Research Topic, we are pleased to present a series of articles that go deeply into certain key aspects of imaging in uveitis. Punctate inner choroiditis (PIC) and multifocal choroiditis (MC) are two types of posterior uveitis that present usually in myopic young women; evaluation of imaging in these diseases may be challenging due to intrinsic retinochoroidal structural abnormalities of these patients that may be confused with inflammatory lesions. Gallego-Pinazo et al. reviewed the multimodal imaging characteristics of pathologic myopia and PIC-MC and underlie the key features that help differentiate them. Another matter of difficulty in these diseases is the diagnosis of macular neovascularization (MNV) that occurs in certain cases. This is a usual complication PIC-MC and its tomographic appearance may be very similar to acute inflammatory lesions. Agarwal et al. in an elegant retrospective study showed that both quantitative and qualitative OCT parameters can aid in the prediction of an underlying MNV in eyes with PIC. These authors have evaluated 35 lesions (35 eyes, 29 patients) and have found that lesions presenting with MNV had more height, width, and volume and more frequently showed the disruptions of Bruch's membrane and ellipsoid zone, fuzziness of outer retina, and hypo-reflective back shadowing than lesions without MNV.

Choroidal assessment is challenging. ICGA is the gold standard for its evaluation but has the shortcomings of the classical angiographic techniques: it is invasive, time-consuming, and non-quantifiable, and assessment of the depth of some lesions is not possible, among others. OCT-A overcomes some of these limitations (although provides their own ones). Tian et al. in their research compared the findings of both techniques (ICGA and SS-OCT-A) in a series of 41 consecutive posterior uveitis patients (68 eyes). Interestingly, 23 (34%) of lesions were visible on OCT-A but not in ICG-A. In turn, of the 45 eyes with visible lesions in ICGA, 22 (49%) and 21 (47%) eyes did not show the areas of flow deficit in CC and choroidal slabs. The size of the lesions was also evaluated. The area of the lesions measured in the ICGA (late frames) was bigger than the area of the lesions detected in CC and choroidal slabs by OCT-A. Imaging may also be useful for discerning the origin of functional deficits. Fuchs' heterochromic uveitis (FHU) is a unilateral, chronic, mostly anterior uveitis with characteristic stellate keratic precipitates that associate posterior subcapsular cataract and ocular hypertension. Not infrequently, the patient complains of poor vision despite visual acuity is good, even 20/20. A possible origin of this symptomatology might be related to the media [mild cataract, keratic precipitates, and vitreal opacities that present in up to 50% of patients (5)] or to retinal abnormalities, for instance, vascular alterations at the macula, which have been shown by means of OCT-A (6). In an interesting work, Zhou et al. analyzed retrospectively the OCT-A macular vascular density (VD), the contrast sensitivity (CS), and the haze of 25 patients with FHU and 30 controls. According to the results, patients with FHU have lower CS, and the opacity of the media (measured by means of haze) would negatively correlate with CS and not with VD. Hence, a retinal origin of the visual dysfunction may be ruled out.

The four articles presented in this Research Topic go deep into some relevant issues in the field of imaging of inflammatory disorders of the eye. We believe that they will be of great interest to clinicians and researchers in this expanding area of knowledge.

## Author contributions

All authors listed have made a substantial, direct, and intellectual contribution to the work and approved it for publication.

## Conflict of interest

The authors declare that the research was conducted in the absence of any commercial or financial relationships that could be construed as a potential conflict of interest.

## Publisher's note

All claims expressed in this article are solely those of the authors and do not necessarily represent those of their affiliated organizations, or those of the publisher, the editors and the reviewers. Any product that may be evaluated in this article, or claim that may be made by its manufacturer, is not guaranteed or endorsed by the publisher.
